# Perioperative and oncologic outcomes of transperitoneal versus retroperitoneal laparoscopic nephroureterectomy for upper urinary tract urothelial carcinoma: a systematic review and pooled analysis of comparative outcomes

**DOI:** 10.1186/s12957-023-03046-1

**Published:** 2023-05-29

**Authors:** Ping-yu Zhu, Li Wang, Kun-peng Li, Shan Yin, Xiao-bin Chen

**Affiliations:** 1 Department of Urology, Affiliated Hospital of North Sichuan Medical College, Nanchong, 637000 China; 2 Department of Urology, Affiliated Hospital of Lanzhou University Second Hospital, Lanzhou, 730030 China

**Keywords:** Nephroureterectomy, Upper urinary tract urothelial carcinoma, Transperitoneal, Retroperitoneal, Meta-analysis

## Abstract

**Background:**

The debate on whether to choose a transperitoneal (TP) or retroperitoneal (RP) approach for treating upper urinary tract urothelial carcinoma (UTUC) with laparoscopic surgery has been drawing attention. This study aimed to systematically review and meta-analyze the existing evidence regarding oncologic and perioperative outcomes of transperitoneal laparoscopic radical nephroureterectomy (TLNU) and retroperitoneal laparoscopic radical nephroureterectomy (RLNU) in managing UTUC.

**Methods:**

A comprehensive literature search was conducted using PubMed, Scopus, Embase, and Google Scholar for identifying randomized controlled trials (RCTs) and observational studies that evaluated the outcomes of TLNU and RLNU for UTUC. Continuous variables were represented by weighted mean difference (WMD) and standard mean difference (SMD), while binary variables were represented by odds ratio (OR), with 95% confidence intervals (CIs). The quality was assessed using the Newcastle–Ottawa scale. A sensitivity analysis was performed to evaluate the robustness of the estimates.

**Result:**

Six observational studies were incorporated into this meta-analysis. The overall TLNU was associated with significantly shorter operating time (WMD − 19.85; 95% CI − 38.03 to − 1.68; *P* = 0.03); longer recovery time of intestinal function (SMD 0.46; 95% CI 0.08 to 0.84; *P* = 0.02). However, the terms of estimated blood loss (WMD − 5.72; 95% CI − 19.6 to − 8.15; *P* = 0.42); length of stay (WMD − 0.35; 95% CI − 1.61 to 0.91; *P* = 0.59), visual analog pain scale (WMD − 0.38; 95% CI − 0.99 to 0.84; *P* = 0.22); drainage duration (WMD − 0.22; 95% CI − 0.61 to 0.17; *P* = 0.26); overall complication rates (OR 1.24; 95% CI 0.58 to 2.63; *P* = 0.58); local recurrence rate (OR 0.6; 95% CI 0.3 to 1.21; *P* = 0.16); distant metastasis (OR 0.94; 95% CI 0.04 to 20.77; *P* = 0.97); 1-year overall survival (OS) (OR 0.45; 95% CI 0.1 to 2.01; *P* = 0.3) showed no difference between TLNU and RLUN.

**Conclusion:**

TLNU provides similar surgical outcomes and oncologic results compared to RLUN; however, TLNU has a shorter procedure time and prolonged intestinal function recovery time. Due to the heterogeneity among the studies, randomized clinical trials with follow-ups in the long term are required to obtain more definite results.

**Trial registration:**

www.crd.york.ac.uk/prospero/, identifier CRD42023388554.

**Supplementary Information:**

The online version contains supplementary material available at 10.1186/s12957-023-03046-1.

## Introduction

Upper urinary tract urothelial carcinoma (UTUC) is a rare, but aggressive genitourinary malignancy. It accounts for approximately 5% of all urothelial cancers, with an increasing incidence rate worldwide in recent years [1]. UTUC is more prevalent in the Chinese population, and its prevalence ranges from 20 to 30%. This could be partially attributed to certain detrimental environmental conditions in China, such as arsenic in drinking water and the prevalent usage of traditional Chinese medicines, which often include herbs containing aristolochic acids [2].

Laparoscopic nephroureterectomy (LNU), removing the kidney and ureter with a bladder cuff, is the standard surgical treatment for UTUC. However, there remain uncertainties regarding the optimal surgical approach for nephroureterectomy. Despite both transperitoneal (TP) and retroperitoneal (RP) approaches being suitable for LNU in patients with UTUC, there is limited evidence regarding the comparative perioperative and oncologic outcomes of the two procedures. The advantages of the transperitoneal approach include easier access to adjacent organs, while the retroperitoneal approach has the benefit of more optimal visualization of the ureter and the renal hilum.

This systematic review and pooled analysis are the first of their kind to compare the outcomes of TLNU and RLNU. Based on the findings of this study, clinicians will be able to make informed decisions regarding the most appropriate surgical approach, thus providing a foundation for future research endeavors in this field.

### Evidence acquisition

#### Search strategy, study selection, and data extraction 

This study was registered with PROSPERO (CRD42023388554).The search strategies, selection criteria, and evidence report were designed following the PRISMA (Preferred Reporting Items for Systematic Review and Meta-Analysis) recommendations (Table S1) [3]. Two researchers (WL and YS) searched for relevant literature in databases, such as PubMed/MEDLINE, Scopus, Google Scholar, and Embase up to January 2023.

The following search string was created by combining patient-related and intervention search terms, including [(upper urinary tract OR urinary bladder OR ureters OR renal pelvis) AND (urothelial carcinoma OR transitional cell carcinoma) AND (transperitoneal OR retroperitoneal) AND (laparoscopic OR laparoscopies OR nephroureterectomy)] (details see Table S2). After screening the titles and abstracts, full texts of potentially relevant studies were examined. Additionally, the references of these studies were also searched manually to ensure no omissions.

The inclusion criteria, based on the PICOS principles, were as follows: P (patients)—all the patients (> 18 years old) were diagnosed with UTUC; I (intervention)—patients who were undergoing TLNU; C (comparator)—RLNU was used as a comparison; O (outcome)—perioperative and pathological outcomes; S (study type)—prospective or retrospective comparative studies. The exclusion criteria were as follows: (1) no comparison between TLNU and RLNU; (2) the type of reviews, case reports, in vitro studies, and commentaries; (3) no available data information. There was no language restriction on the relevant content studied.

Two independent researchers, namely XB and KP extracted data from the eligible documents according to a pre-set Excel table. The relevant information was as follows: (1) demographic and clinical features: author, year of publication, country, number of participants, gender, age, body mass index (BMI), American Society of Anesthesiologists (ASA) score, tumor laterality, tumor location, tumor stage, and grade, adjuvant therapy and follow-up time. (2) Perioperative outcome: operation time, hospitalization time, intestinal function recovery time (defined as first postoperative expelling gas or defecation) [4], intraoperative blood loss, transfusion rate, visual analog scale pain score, drainage tube removal time, and postoperative complications based on Clavien-Dingo grade classification [5]. (3) Oncologic outcomes: local recurrence rate, distant metastasis rate, and the overall survival (OS) rate one year after surgery.

### Quality and risk of bias assessment

The Oxford Level of Evidence Working Group 2011 was used to set the level of evidence [6], and the Newcastle–Ottawa scale (NOS) for nonrandomized controlled trials was adopted to evaluate the included studies’ quality. Scores below 5 were deemed to be of low quality, scores between 6 and 7 of intermediate quality, and scores between 8 and 9 of high quality [7]. ROBINS-I (Risk of Bias In Non-randomized Studies-of Interventions) was used to assess bias risk in non-randomized studies in meta-analysis [8]. It consisted of nine domains of bias, such as randomization, exposure assessment, potential confounders, selection, attrition of participants, reporting by investigators, comparison of groups, the timing of assessments, control of feedback, and enforced study, which were used to evaluate the bias risk for each study and assess the reliability of key outcomes.

### Statistical analysis

A meta-analysis using Cochrane Collaborative RevMan5.4 software and Stata 14.0 was conducted by adopting the random-effects model. Continuous variables were evaluated using the weighted mean difference (WMD) and standardized mean difference (SMD), while odds ratio (OR) was used to assess binary variables, with 95% confidence intervals (CIs). The *I*^2^ test was used to measure the heterogeneity among studies with statistical significance set at *P* < 0.05. Conflicting opinions between reviewers were reconciled by coming to a mutual agreement.

### Publication bias

The test power was inadequate due to the small number of studies (< 10) included; thus, preventing us from evaluating the presence of publication bias [9, 10].

## Results

### Baseline characteristics

**Fig. 1:**
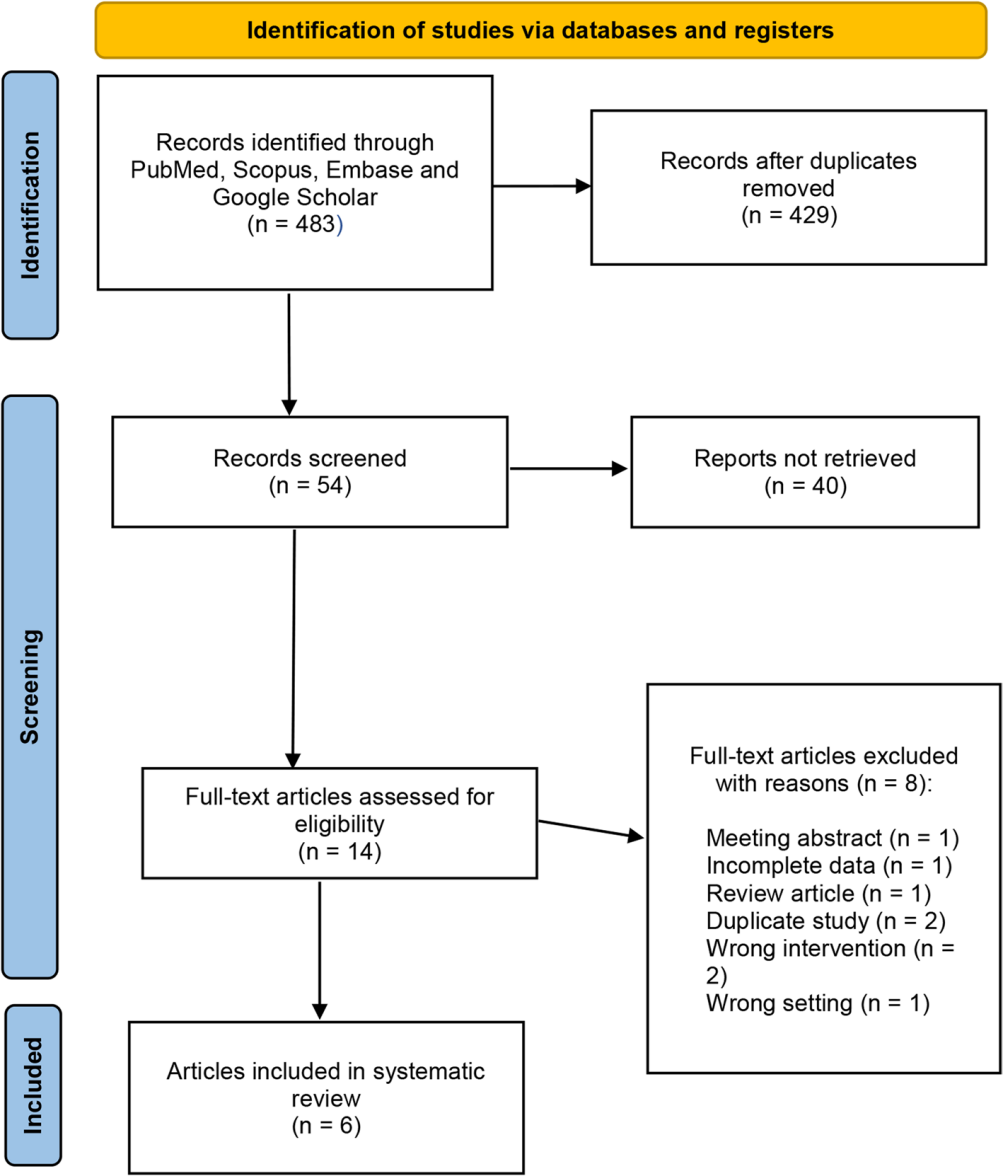
PRISMA flowchart

After our initial search, which identified 110 studies with 596 patients (261 TLNU vs. 335 RLNU). Ultimately, we determined six studies published between 2016 and 2022 that passed our initial title and abstract screening. One study was a prospective non-randomized controlled trial [11], while the other five studies were retrospective observational case–control studies [12–16]. One of the trials is a multi-center study [13]. The literature screening process is illustrated in Fig. 1. The summary of the content related to demographics and clinical characteristics is presented in Table 1. All the patients included in this study were from China. The outcomes related to surgery are shown in Table S3. The pathological parameters of the patients are summarized in Table S4. Table S5 showed that no statistically significant differences were found between the two groups in terms of gender (*P* = 0.86), BMI (*P* = 0.14), age (*P* = 0.71), tumor laterality (*P* = 0.16), and ASA score (< 3) (*P* = 0.79).

**Table 1:** The baseline patient characteristics and methodological assessments

References	Ye et al. (2020) [11]		Zhang et al. (2019) [12]		Wu et al. (2019) [13]		Wang et al. (2022) [14]		Liu et al. (2016) [15]		Chen et al. (2019) [16]	
	TLNU	RLNU	TLNU	RLNU	TLNU	RLNU	TLNU	RLNU	TLNU	RLNU	TLNU	RLNU
Country	China		China		China		China		China		China	
Number of patients (*n*)	24	24	45	44	94	172	28	12	34	34	36	49
Design	Prospective		Retrospective		Retrospective		Retrospective		Retrospective		Retrospective	
Age (years)	64.6(13.3)	63.9 (10.2)	65.8(6.06)	65.68(3.13)	66(9.2)	67.1(11.5)	70.86(7)	68.5(8.52)	50.5(9.8)	52.2(10.2)	58.8(7.3)	57.6(8.5)
BMI (kg/m^2^)	21.9(1.4)	22.5(1.3)	NA		23.9(3.4)	24.4(2.9)	24.74(3.37)	24.88(2.59)	23.4(2.1)	23.1(2.5)	NA	
Male/female	15/9	13/11	24/21	23/21	53/41	92/80	19/9	8/4	21/13	24/10	16/20	28/21
ASA score	I:16; II:8	I:18; II:6	NA		I:9; II:59; III:11	I:6; II:112; III:28	NA		I–II:25; III–IV:9	I–II:29; III–IV:5	NA	
Tumor site (Lt/Rt)	14/10	16/8	26/19	23/21	39/55	107/65	17/11	8/4	19/15	19/15	NA	
Tumor location	Pelvicalyceal:13 Pelvicalyceal–ureteral:2 Ureteral:9	Pelvicalyceal:15 Pelvicalyceal–ureteral:1 Ureteral:8	Pelvis:31 Ureter:14	Pelvis: 29 Ureter: 15	NA		NA		Pelvicalyceal: 22 Pelvicalyceal–ureteral: 2; Ureteral: 10	Pelvicalyceal:24 Pelvicalyceal–ureteral: 4 Ureteral:6	Pelvicalyceal: 33 Ureteral: 3	Pelvicalyceal: 41 Ureteral: 8
Lymph node invasion (*n*)	0	0	NA		6	7	1	0	NA		0	0
lymph node Dissection	No		NA		Yes		Yes		NA		No	
Adjuvant therapy	NA		Chemotherapy (pirarubicin): All		NA		IBI of pirarubicin: 3; Chemotherapy (GC): 21; ICT: 5	IBI of pirarubicin: 12; Chemotherapy (GC): 9; ICT: 0	IBI of pirarubicin: all		Bladder infusion chemotherapy: all	
Type of BCE	Laparoscope	Open surgery	Laparoscope	Open surgery	Laparoscope	Open surgery	Laparoscope	Laparoscope	Laparoscope	Open surgery	Laparoscope	Open surgery
Follow-up (month)	NA		1 to 88		NA		12		20.3(5.4)	22.1(6.8)	6 to 60	
Quality score	7		7		8		8		7		7	
Study quality	Low		Moderate		Low		Moderate		Moderate		Low	

*TLNU* Transperitoneal laparoscopic radical nephroureterectomy, *RLNU* Retroperitoneal laparoscopic radical nephroureterectomy, *BMI* Body mass index, *ASA* American Society of Anesthesiologists, *IBI* Immediate bladder irrigation, *GC* Gemcitabine plus cisplatin chemotherapy, *ICT* Immune checkpoint therapy, *BCE* Bladder cuff excision, *NA* Not available, *Mean (SD)* The quality of the included studies was determined using Grading of Recommendations, Assessment, Development and Evaluation (GRADE) Working Group quality assessment

### Assessment of quality

The research quality was assessed by the GRADE Working Group according to their suggestion, evaluation, development, and grading, and the research was found to be at a medium or lower level. Details regarding the NOS scoring can be found in Table S6.

### Outcome analysis

#### Surgical effectiveness

**Fig. 2:**
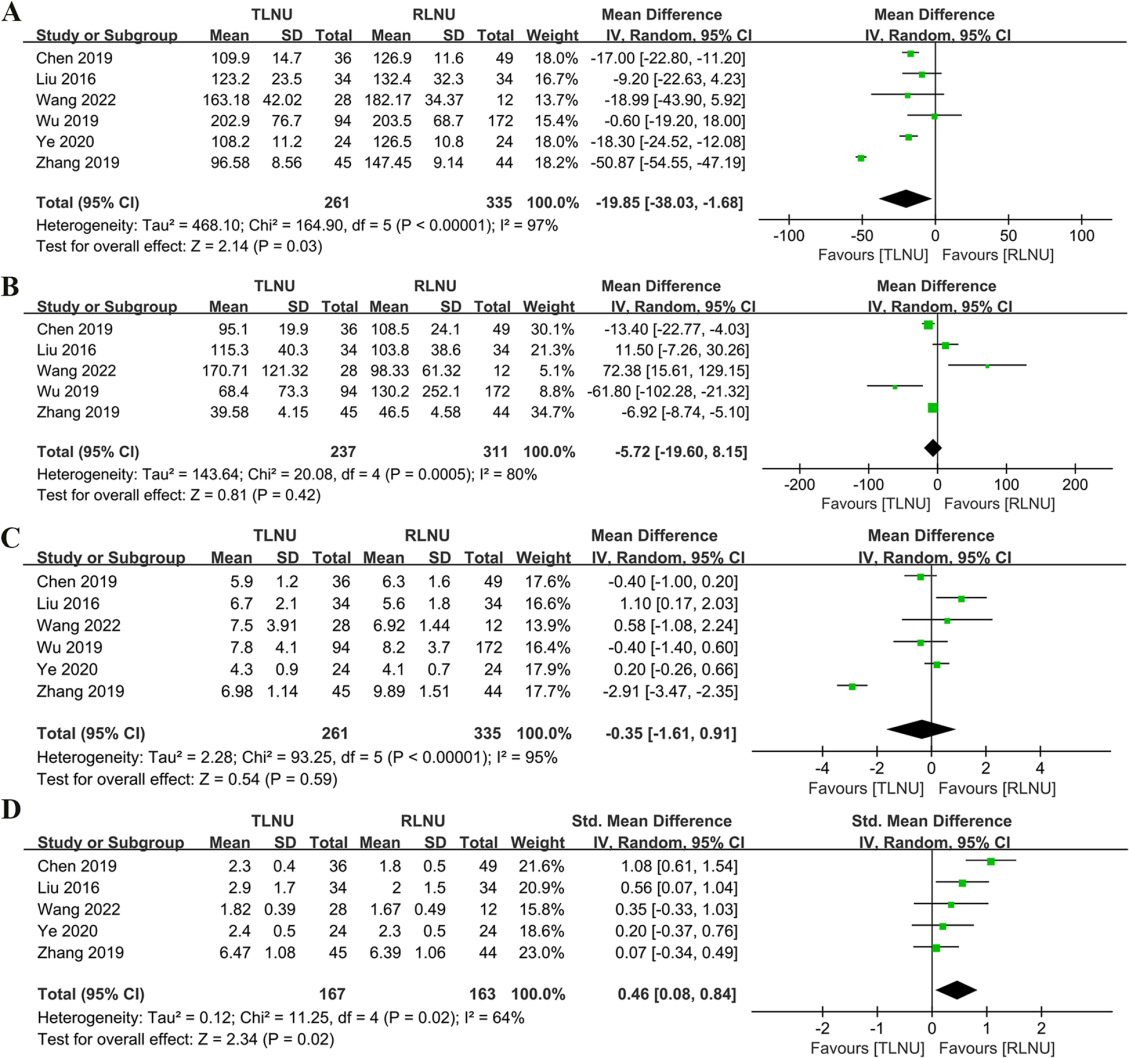
Forest plot of perioperative outcomes: **A** operative time (min); **B** estimated blood loss (ml); **C** length of stay (day); **D** recovery of bowel function (day)

A forest plot of the cumulative analysis of 596 patients (261 TLNU vs. 335 RLNU) reported in six studies showed a statistically significant shorter operative time for the TLNU group when compared with the RLNU group (WMD − 19.85 min; 95% CI − 38.83 to − 1.68; *P* = 0.03) (Fig. 2A). The meta-analysis results of the five studies (237 TLNU vs. 311 RLNU) showed that there was no significant difference between TLNU and RLNU in estimating the blood loss amount (WMD − 5.72 ml; 95% CI − 19.6 to − 8.15; *P* = 0.42) (Fig. 2B). Accumulative analysis showed no obvious difference in the length of hospital stay between the two surgical paths (WMD − 0.35 day; 95% CI − 1.61 to 0.91; *P* = 0.59) (Fig. 2C). However, RLNU showed better postoperative intestinal function recovery time than TLNU, with a statistically significant difference (SMD 0.46 day; 95% CI 0.08 to 0.84; *P* = 0.02) (Fig. 2D).

**Fig. 3:**
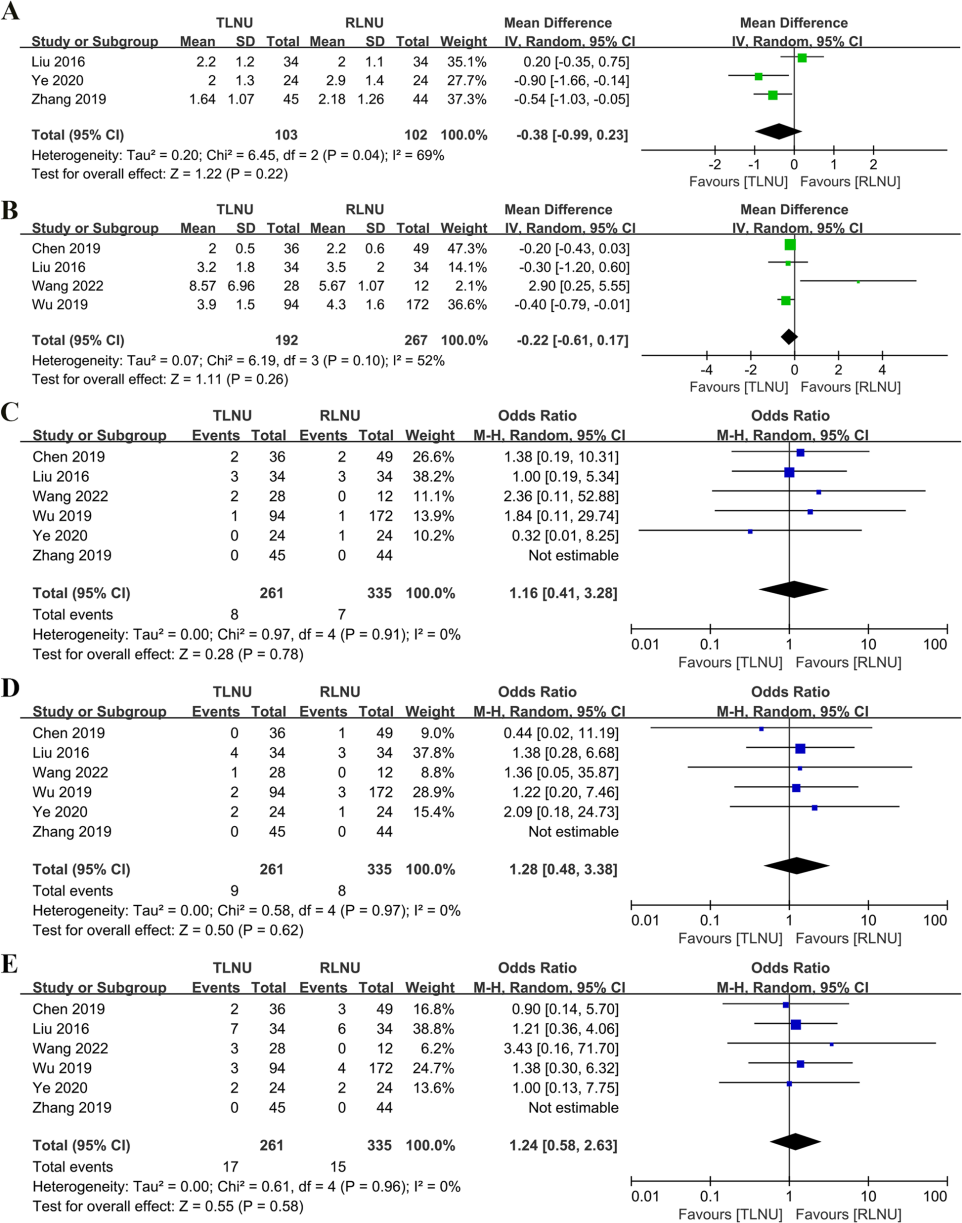
Forest plot of perioperative outcomes: **A** visual analog pain scale; **B** drainage duration (day); **C** Clavien grade I; **D** Clavien grade II; **E** overall postoperative complications

Pooled analysis showed that there was no statistically significant difference between TLNU and RLNU in terms of visual pain simulation scores, indicating a similar performance (WMD − 0.38; 95% CI − 0.99 to 0.84; *P* = 0.22) (Fig. 3A). The accumulative analysis results of four studies [13–16] showed no significant difference between the two surgical approaches in terms of postoperative drainage time (WMD − 0.22; 95% CI − 0.61 to 0.17; *P* = 0.26) (Fig. 3B).

#### Complications

Six studies [11–16] reported postoperative complications, such as postoperative bleeding, fever, intestinal obstruction, etc. No statistically significant difference was observed between the two surgical approaches in the incidence of Clavien-Dindo grade I (*P* = 0.78) and grade II (*P* = 0.62) (Fig. 3C, D). Overall complication rates were 6.51% (17/261) and 4.47% (15/335) in the TLNU and RLNU groups, respectively (OR 1.24; 95% CI 0.28 to 2.63; *P* = 0.58) (Fig. 3E).

#### Oncologic outcomes

**Fig. 4:**
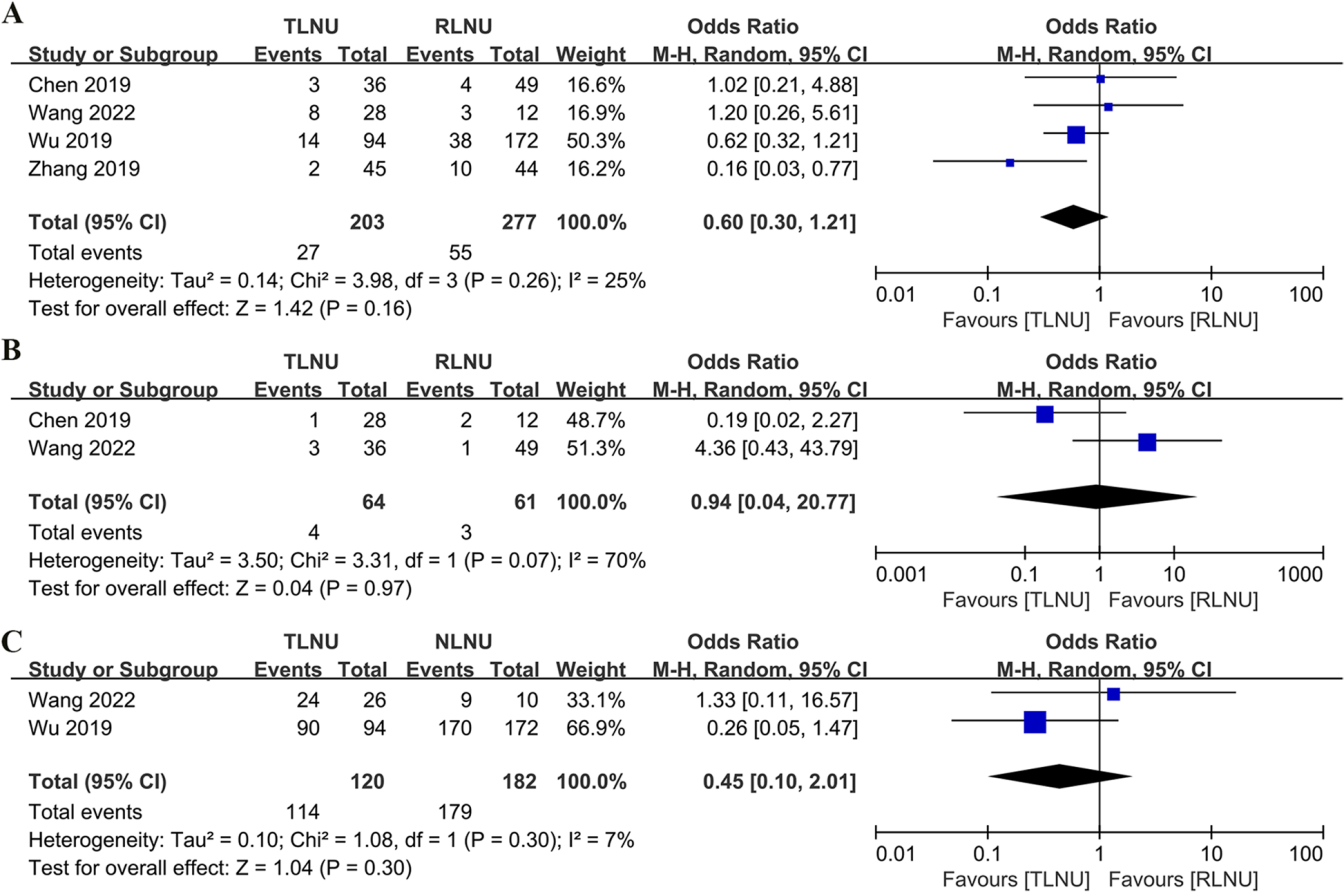
Forest plot of oncological outcomes: **A** local recurrence; **B** distant metastasis; **C** 1-year over survival

Four studies [12–14, 16] describing postoperative local recurrence were cumulatively analyzed that showed similar local recurrence rates for TLNU and RTNU with no statistically significant difference (OR 0.6; 95% CI 0.3 to 1.21; *P* = 0.16) (Fig. 4A). Similarly, TLNU and RLNU had similar outcomes (OR 0.94; 95% CI 0.04 to 20.77; *P* = 0.97) for postoperative distant metastasis (Fig. 4B). Two studies [13, 14] reported the overall one-year survival rate of patients after surgery. The results of the analysis showed that there was no statistically significant difference in the 1-year overall survival rate between the two routes, via the abdominal or posterior abdominal routes (OR 0.45; 95% CI: 0.1 to 2.01; *P* = 0.3) (Fig. 4C).

### Sensitivity analysis and risk of bias

**Fig. 5:**
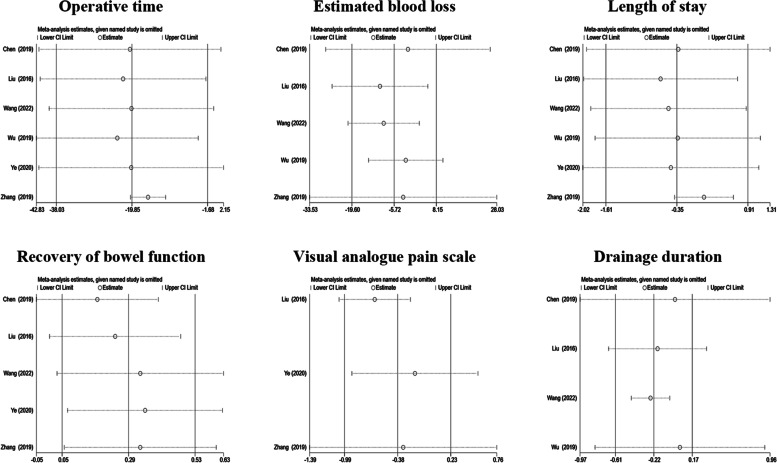
Sensitivity analysis of surgical outcome

Sensitivity analysis was performed for studies with high heterogeneity (*I*^2^ > 40%) to strengthen the reliability of the analysis. Studies were individually deleted, and the pooled effect value was recalculated. Sensitivity analysis cannot be conducted when there are three or fewer studies to compare [17]. All the newly pooled effect values remained unchanged after the deletion of any study (Fig. 5). The results of the ROBINS-I assessment of publication bias indicate that all comparative studies have a moderate risk of bias (Table S7).

#### Heterogeneity

Most of the research results show moderate to high heterogeneity, such as operative time, estimated blood loss, hospital stay, bowel function recovery time, the visual analog scale of pain, drainage time, and distant metastasis rate. Certainly, the statistical bias caused by small sample size studies cannot be ignored [17].

## Discussion

This is the first clinical data-based comparative study on the efficacy and safety of LNU through the TP and RP approaches for treating UTUC, and its findings warrant further exploration.

### Surgical outcomes

The operation time for treating UTUC via the TP approach is shorter compared to the RP approach. We suggest that the TLNU has a larger operative space. Furthermore, the surgeons are more familiar with the anatomy and positioning via the transperitoneal approach. However, this contradicts the previous studies [18] suggesting laparoscopic nephrectomy via the retroperitoneal approach can directly enter the kidney, thereby reducing the operation time by avoiding mobilization of other organs and ureters around the intestine and kidney. It is noteworthy that while undergoing RLNU, the patient's position needs to be changed, which would affect the calculation of the actual operation time. Appropriate intraoperative posture can better coordinate the cooperation of surgeons and assistants and reduce the operation time [19].

Recently, Wu et al. [20] evaluated the outcomes of a complete RLNU with bladder cuff excision (BCE), which had a median operation time of 110 min. Miki et al. [21] performed a complete laparoscopic nephrectomy in the prone position in 20 patients with UTUC, with an average operation time of 234 min. In the future, this approach could become the standard of care for UTUC patients. Previous studies on minimally invasive urologic procedures have demonstrated the numerous advantages of the RP approach over the TP approach, such as better intraoperative organ protection, early renal arterial control, and reduced intraoperative bleeding due to minimized intraoperative dissection [22]. However, our cumulative analysis indicated that the estimated blood loss of both TLNU and RLNU were comparable.

RLNU performed under retroperitoneal laparoscopy avoided intestinal interference, reduced the incidence of gastrointestinal dysfunction and intestinal obstruction, and enabled patients to expel gas and defecate earlier, thus accelerating postoperative recovery. Our cumulative analysis also confirmed this result (*P* = 0.02). Theoretically, this result would indirectly affect the patients’ length of hospital stay [15]. However, our small-sample pooled analysis showed no statistically significant differences between TLNU and RLNU (*P* = 0.59). Moreover, the length of hospital stay was mainly determined by the surgeon’s recovery concept and the hospital’s capacity [23]. Compared with RLNU, TLNU has similar outcomes in the visual analog scale of pain and postoperative drain retention time. Ye et al. [11] suggested that patients undergoing RLNU experience more severe pain, attributed to the cutting of the lateral abdominal muscles, overextension in the lateral recumbent position during the procedure, and the potential for large area damage caused by the removal of the peritoneum and adipose tissue. Peng et al. showed that transperitoneal pneumoperitoneum has a higher central venous pressure than retroperitoneal pneumoperitoneum [24]. In addition, compared with high pneumoperitoneum pressure, low pressure can reduce the incidence of intestinal obstruction and postoperative pain in minimally invasive urological surgery. while the estimated blood loss was comparable [25, 26].The surgeon’s experience and skills would affect the postoperative drainage duration. Caution should be exercised when interpreting the results of this research due to its heterogeneity.

### Complications

Analysis of current studies revealed that TLNU and RLNU had no statistical difference in terms of overall complication rates (6.51% vs. 4.47%). No major complications (Clavien-Dindo grade ≥ III) were reported in any of the studies. Ren et al. [22] further revealed that the overall complication rates between TP and RP routes of laparoscopic radical nephrectomy were similar (*P* = 0.406). In addition, Liu et al. [27] compared laparoscopic and open surgery for UTUC and found that RLNU had lower overall complication rates than open surgery (*P* < 0.01). LNU might reduce surgical trauma and increase the postoperative recovery rate. However, some recent studies have suggested that it might lead to chronic pain and fatigue in patients due to bladder suspension tightening and functional impairment. Moreover, LNU might cause weight loss in patients.

### Oncologic outcomes

Our study focused on local recurrence rates, distant metastases, and 1-year overall survival. Cumulative analysis showed that TLNU and RLNU had similar prognostic outcomes. The incidence of port metastasis and the role of the pneumoperitoneal hyperbaric environment in tumor cell seeding have been controversial in laparoscopic surgery. High-grade invasive tumors have been reported to be associated with cases of peritoneal cancer dissemination or early metastases at unusual metastatic sites [28]. Morselli’s [29] study of three centers revealed only three cases of local recurrence in laparoscopic radical nephroureterectomy (RNU) compared to open surgery, with comparable recurrence rates in both groups (*P* = 0.594), and notably in regression analysis, tumor stage, and surgical access were independent predictors. Whether to perform ureteroscopy before RNU is still controversial. Although there is a clear relationship between ureteroscopy and the increased risk of intravesical recurrence (IVR), it does not affect the survival results [30]. Hemal et al. [31] performed RLNU-BCE on 21 patients. The results showed no local recurrence; however, the bladder recurrence rate was 9.52%. Certainly, immediate postoperative chemotherapy infusion in surgical patients significantly reduces bladder tumor recurrence. Second, bladder cuff and distal ureter management might play a critical oncological role. When comparing the outcomes of extravesical, transvesical, and endoscopic approaches in a retrospective study of 2681 patients who underwent RNU, no differences in non-bladder recurrence or survival were found [32]. According to Yuan et al. [33], the following significant risk factors were related to subsequent intravesical recurrence after RNU: female patients; ureteral tumor; larger tumor; Tis, Ta, and T1; and the history of bladder cancer. Kim’s [34] analysis of 743 patients revealed that the RP approach is associated with better progression-free survival (PFS) than the TP approach in UTUC patients, while cancer-specific survival (CCS) and OS were equivalent between the two nephroureterectomy groups. Moreover, the surgical technique was not considered to be related to PFS, CSS, or OS. RP procedure offers the advantage of earlier exposure to the kidney and ureter than TP, which can reduce the risk of tumor cells migrating to the urinary system or bloodstream. A study from the seer database showed that compared with pure UTUC patients, those with histological variants often had advanced disease and higher CSS at all stages, However, in T1-2 stage disease, RNU results in similar survival in squamous cell carcinoma (SCC) or adenocarcinoma versus UTUC [35]. Neoadjuvant systemic therapy, specifically neoadjuvant chemotherapy (NAC), has garnered increasing attention. A recent comprehensive assessment revealed that NAC leads to enhanced survival rates and improved pathological responses when compared to surgery alone. However, it does not offer any significant additional benefits compared to the combination of surgery followed by adjuvant chemotherapy [36]. In metastatic UTUC, chemotherapy and radical nephrectomy are protective factors for higher survival, while patients with liver metastases often predict a poorer prognosis, although platinum-based combination chemotherapy is currently used as a first-line treatment option and immunotherapy with PD-1/PD-L1 pathway inhibition has been shown to be safe and effective in the systemic management of metastatic UTUC [35, 37–39].

An updated meta-analysis concluded that LRNU and open surgery offer equivalent cancer control outcomes in patients with UTUC, including those with locally advanced disease [40]. Robot-assisted nephroureterectomy (RANU) is a novel treatment for UTUC. It is becoming increasingly popular because it offers the advantages of good stereotactic accuracy, controllability, and repeatability. RANU can offer fewer complications and shorter hospital stays than laparoscopic surgery [41]. the robotic platform might allow easier achievement of a watertight bladder closure. This appears to be more conducive to the perioperative perfusion of mitomycin [30]. In addition, UTUC ranks as the third most prevalent malignancy observed in individuals with Lynch syndrome (LS). Alarmingly, up to 21% of newly diagnosed UTUC cases may harbor undetected LS as the fundamental etiology. Consequently, future endeavors should focus on advancing diagnostic techniques and screening protocols to address this pressing concern [42].

### Limitations

Limitations of our study should also be acknowledged. Firstly, the sample size of our research population was small, and all participants were from China. This could have led to selection bias. Moreover, the documents included were retrospective controlled studies with relatively low overall evidence quality. Secondly, although the sensitivity analysis explained some of the heterogeneity, subgroup analysis was not performed due to a lack of effective data. Hence, results should be interpreted cautiously. Finally, some research results inevitably have variations due to the differences in hospital equipment and the skill level of the doctors performing the surgery.

## Conclusions

Our analysis suggests that TLNU and RLNU have similar surgical and oncological outcomes. Although TLNU has a shorter operative time, its intestinal recovery time is longer than RLNU. Despite the high heterogeneity among the studies, additional evidence is required to verify the robustness of the results, preferably from the long-term follow-up of prospective randomized clinical trials.

## Supplementary Information

**Additional file 1:** **Table S1.** PRISMA 2020 Checklist. **Table S2.** Search strategy employed. **Table S3**. Surgical outcomes. **Table S4.** Pathological parameters. **Table S5.** Comparison of baseline demographics. **Table S6.** Study quality based on the Newcastle-Ottawa Scale. **Table S7.** The ROBINS-I for risk of bias.

## Data Availability

The article and the supplementary material contain all the datasets that were produced by this investigation.

## Abbreviations

TP: Transperitoneal
RP: Retroperitoneal
UTUC: Urinary tract urothelial carcinoma
TLNU: Transperitoneal laparoscopic radical nephroureterectomy
RLNU: Retroperitoneal laparoscopic radical nephroureterectomy
RCTs: Randomized controlled trials
WMD: Weighted mean difference
SMD: Standard mean difference
OR: Odds ratio
CIs: Confidence intervals
OS: Overall survival
LNU: Laparoscopic nephroureterectomy
PRISMA: Preferred Reporting Items for Systematic Review and Meta-Analysis
BMI: Body mass index
ASA: American Society of Anesthesiologists
NOS: Newcastle-Ottawa Scale
ROBINS-I: Risk of Bias In Non-randomised Studies of Interventions
BCE: Bladder cuff excision
RNU: Radical nephroureterectomy
IVR: Intravesical recurrence
PFS: Progression-free survival
CCS: Cancer-specific survival
SCC: Squamous cell carcinoma
RANU: Robot-assisted nephroureterectomy
NAC: Neoadjuvant chemotherapy
LS: Lynch syndrome
